# Molecular detection and targeting of EWSR1 fusion transcripts in soft tissue tumors

**DOI:** 10.1007/s12032-012-0412-8

**Published:** 2013-01-18

**Authors:** Monica Cantile, Laura Marra, Renato Franco, Paolo Ascierto, Giuseppina Liguori, Annarosaria De Chiara, Gerardo Botti

**Affiliations:** 1 Pathology Unit, National Cancer Institute “Fondazione G. Pascale”, Via Mariano Semmola, 80131 Naples, Italy; 2 Unit of Medical Oncology and Innovative Therapies, National Cancer Institute “Fondazione G. Pascale”, Via Mariano Semmola, 80131 Naples, Italy

**Keywords:** EWSR1 partners, Fusion transcript detection, Fusion transcript inhibition

## Abstract

Soft tissue tumors are a heterogeneous group of tumors, traditionally classified according to morphology and histogenesis. Molecular classification divides sarcomas into two main categories: (a) sarcomas with specific genetic alterations and (b) sarcomas showing multiple complex karyotypic abnormalities without any specific pattern. Most chromosomal alterations are represented by translocations which are increasingly detected. The identification of fusion transcripts, in fact, not only support the diagnosis but also provides the basis for the development of new therapeutic strategies aimed at blocking aberrant activity of the chimeric proteins. One of the genes most susceptible to breakage/translocation in soft tissue tumors is represented by Ewing sarcoma breakpoint region 1 (EWSR1). This gene has a large number of fusion partners, mainly associated with the pathogenesis of Ewing’s sarcoma but with other soft tissue tumors too. In this review, we illustrate the characteristics of this gene/protein, both in normal cellular physiology and in carcinogenesis. We describe the different fusion partners of EWSR1, the molecular pathways in which is involved and the main molecular biology techniques for the identification of fusion transcripts and for their inhibition.

## EWS gene and protein structure

**Fig. 1:**
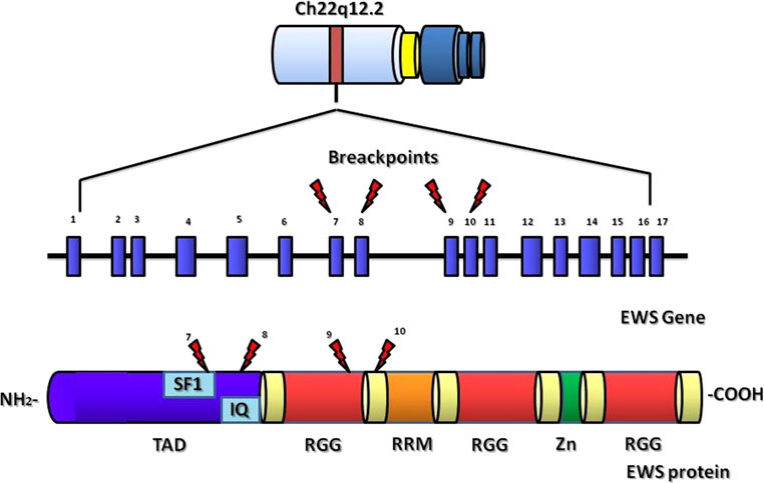
Schematic representation of EWS gene and protein domains with the main breakpoints illustrations

EWR1 is located on 22q12.2 chromosome and spans about 40 kb of DNA with 17 exons. The first 7 exons encode the N-terminal domain and exons 11, 12 and 13 encode the putative RNA-binding domain. The protein consists of 656 amino acids and 68.5 kDa of size and is composed of several domains. TAD domain is a transactivation domain that contains a consensus sequence repeated SYGQ and two auto regulation regions, IQ and SF1. The domain IQ binds to calmodulin, inhibiting phosphorylation by protein kinase C (PKC) and favoring the activation of EWS. On the contrary, the binding with SF1 (splicing factor 1) inhibits the activation domain TAD, negatively regulating target genes of EWS. There are also three RGG domains rich in arginine and glycine, a domain RBD that binds single filament of RNA and DNA and a ring finger domain similar to the RAN-BP2 [1]. Regarding EWSR1 breakpoints, not all the regions composing the protein are susceptible to breakage in soft tissue tumors [2]. The areas mainly involved in translocation are EWSR1 exons 7 and 8, coding for the region SYGQ and exons 9 and 10, associated with regions protein RGG [3] (Fig. 1).

## EWS protein in normal cell physiology

EWS is a member of the TET (Tls, EWSR1, TafII) family of RNA-binding proteins, with FUS (TLS) and TAF15 (TAFII68). The codified protein is involved in transcriptional regulation for specific genes and in mRNA splicing. In particular, EWSR1 plays a role in transcription initiation. In fact, EWSR1 is able to link with the basal transcription factor TFIID and RNA polymerase II complex.

In normal cells, EWSR1 is phosphorylated by PRKC (protein kinase C) although its IQ domain, which inhibits RNA-binding EWSR1. EWSR1 is required for proper localization of aurora B during mitosis, also maintaining mitotic spindle integrity [4]. EWSR1 also associates with EP300 and CREBBP acting as coactivator of CREBBP-dependent transcription factors. EWSR1-EP300/CREBBP mediates FOS activation, as well as HNF4 genes activation [5, 6]. EWSR1 activates other transcription factors such as POU4F1 (or BRN3A, 13q13) [7], and POU5F1 (or OCT4, 6p21) genes which regulate differentiation of neuronal cells [7, 8]. EWSR1 and CCNL1 (cyclin L1) are also interacting partners of TFIP11 (tuftelin-interacting protein 11), a protein functionally related to the spliceosome and involved in pre-mRNA splicing [9]. In normal cell physiology, EWSR1 is required for cell survival in the central nervous system [4] and performs important functions in the regulation of genomic integrity and in RNA and microRNA’s maturation processes.

Regarding its localization, EWSR1 is ubiquity expressed [10, 11] with prevalent localization in the nucleus and more rarely in the cytoplasm and various subcellular compartments, depending on the methylation state of its RNA-binding domain [12]. Localization of EWSR1 in different subcellular compartments reflects its dynamic distribution during cell cycle [13].

## EWS translocation partners in cancer

Several gene families, mainly encoding for transcriptional regulators, can translocate with EWSR1. The chimeric proteins have an aberrant activity interfering with different molecular pathways crucial for cell growth, differentiation and proliferation. These altered interactions are often responsible for the pathogenesis of soft tissue tumors.

### ETS transcription factor family

**Fig. 2:**
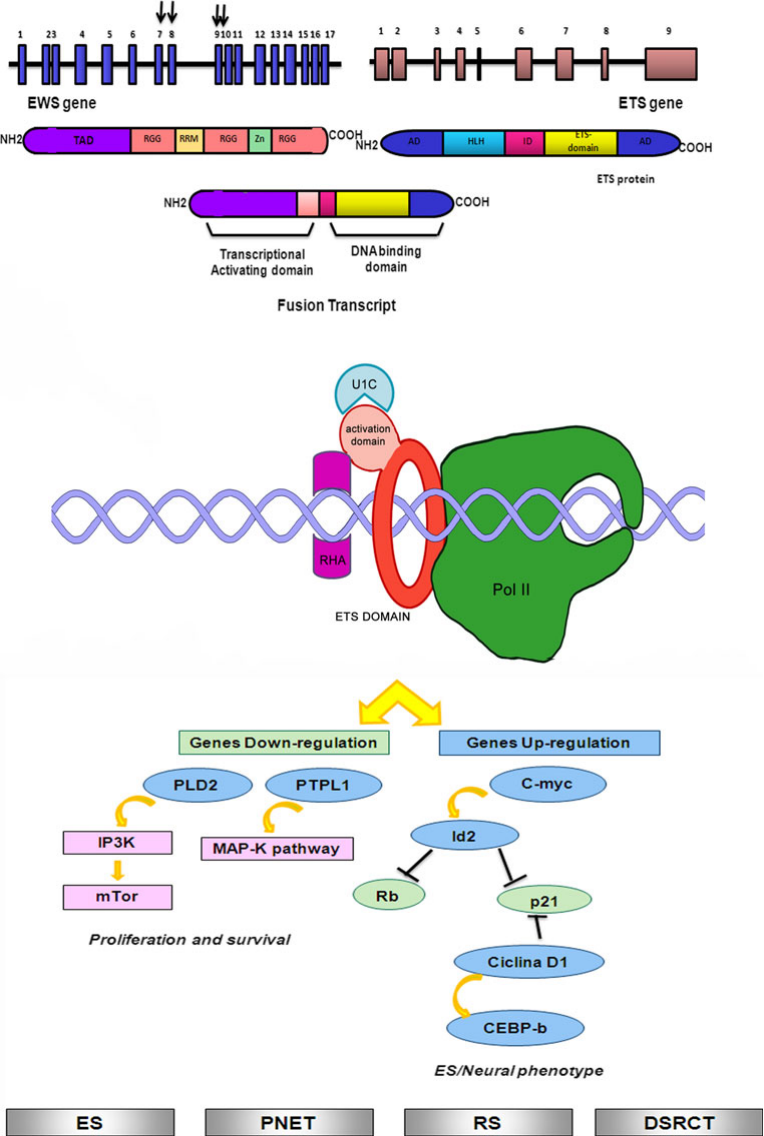
Schematic representation of EWS translocation with ETS-genes family fusion partners: interaction with transcriptional complex on DNA and illustration of the main molecular pathways deregulated. *Gray squares* indicate the soft tissue tumors characterized by EWS/ETS translocations (ES: Ewing sarcoma; PNET: Primitive neuroectodermal tumor; RS: Rhabdomyosarcoma; DSRCT: Desmoplastic small-round-cell-tumor)

The ETS (E twenty-six) family (Fig. 2) is one of the largest families of transcriptional factors involved in a wide variety of functions, including the regulation of cell differentiation, cell-cycle control, cell migration, cell proliferation, apoptosis and angiogenesis. All ETS family members are identified through a highly conserved DNA-binding domain, the ETS domain, which is a winged helix-turn-helix structure. The ETS family ETS domain is also involved in protein–protein interactions.

#### EWSR1/FLI1 (t(11;22)(q24;q12))

Fli1 (Friend leukemia integration 1 transcription factor) was identified in the mouse genome as a viral integration site common to 2 retroviruses involved in virus-induced leukemias and lymphomas. The mouse Fli1 region is close to the centromere of chromosome 9 [14], while human FLI1 gene is located on chromosome 11q23-q24. It lies on a fragment flanked on the centromeric side, by the translocation breakpoint in acute lymphoblastic leukemia-associated t(4;11)(q21;q23) [15] and on the telomeric side in Ewing sarcoma-associated t(11;22)(q24;q12) breakpoint [16]. Several breakpoints have been described for EWSR1 and FLI1 in soft tissue tumors. The most common are EWSR1 exon 7 and FLI1 exon 6 (defined as Type I), EWSR1 exon 7 and FLI1 exon 5 (defined as Type II), EWSR1 exon 10 and FLI1 exon 6 (defined as Type III), and EWSR1 exon 7 and FLI1 exon 7 (defined as Type IV). Many other translocations have been described, such as EWSR1 exon 7 and FLI1 exon 8, EWSR1 exon 8 and FLI1 exon 6, EWSR1 exon 9 and FLI1 exon 4, EWSR1 exon 9 and FLI1 exon 7, EWSR1 exon 10 and FLI1 exon 5 and EWSR1 exon 10 and FLI1 exon 8 [3]. The fusion transcript EWSR1/FLI1, as well as many other chimeric proteins, interferes with several molecular pathways, influencing the development and progression of this tumor. In particular, the interaction of the chimeric transcript with important mediators of cell cycle has been described. EWSR1/FLI1 results in an upregulation of c-Myc and a downregulation of p57KIP2 [17]. In addition, the fusion transcript is able to inhibit the transcriptional activity of p53 [18]. EWSR1/FLI1 also determines an upregulation of GLI, altering the molecular pathways associated with it [19]. It has been recently demonstrated, using cellular models, that EWSR1/FLI1 is able to block the ability of Runx2 in order to induce osteoblast differentiation, responsible of Ewing tumor pathogenesis [20]. Recent studies have showed the strong association between chimeric transcripts and microRNA activity. EWS/FLI1 is able to modulate miRNA 145 controlling cell differentiation [21]. Additionally, deregulation of let7a is strongly related to EWS/FLI1 production [22]. The fusion transcript EWSR1/FLI1 is present in more than 90 % of the Ewing sarcomas, and in the related group of peripheral primitive neuroectodermal tumor (pPNET) [23], in rhabdomyosarcoma [24], in neuroblastoma [25] and in giant cell tumor of bone [26].

#### EWS/ERG (t(21;22)(q22;q12))

The transcription factor Erg is essential for definitive hematopoiesis and for the function of adult hematopoietic stem cells [27]. Chromosomal rearrangements involving ERG are found in acute myeloid leukemia, acute lymphoblastic leukemia, Ewing’s sarcoma and more than half of all prostate cancers, but the normal physiological function of Erg is unknown. The chromosomal translocation t(21;22)(q22;q12) has been described in approximately 10 % of Ewing’s sarcoma tumors. ERG shares 68 % overall amino acid identity with FLI and 98 % identity within their ETS DNA-binding domains. Considering the structural similarities of EWS/FLI and EWS/ERG fusions, it is likely that the two proteins act in order to deregulate similar target genes in Ewing’s sarcoma. In fact, a retrospective study comparing EWS/ERG Ewing’s sarcoma cases with EWS/FLI cases revealed no significant differences in pathological and clinical characteristics as well as overall survival. Several breakpoints have been described for EWSR1 and ERG in soft tissue tumors. The most common is EWS exon 7, which translocates to ERG exon 6, 7 and 9 [28]. Although the chimeric transcript EWSR1/ERG is able to interfere with different cellular pathways, for example, together with other EWS partners, it can suppress TGF-beta R activity [29]. Several ECM molecules are modulated by EWS/ERG, such as collagen COL11A2 [30], and laminin beta3 [31]. This fusion transcript can also be present in other peripheral primitive neuroectodermal tumor (pPNET) [32] and in desmoplastic small round cell tumor (DSRCT) [33].

#### EWSR1/FEV t(2;22)(q33;q12); EWSR1/ETV1 t(7;22)(p22;q12); EWSR1/ETV4 t(17;22)(q21;q12)

EWSR1/ETV1, EWSR1/ETV4 and EWSR1/FEV fusions occur in <1 % of Ewing sarcomas. FLI, ERG and FEV share 87 % identity and 98 % similarity, while ETV1 and ETV4 share 96 % identity and 100 % similarity in their DNA-binding domains. Exon 7 of EWSR1 gene can translocate to FEV exon 2, ETV1 exon 11 and ETV4 exon 9 [34–36]. Many studies showed that EWSR1/FEV, EWSR1/FLI and EWSR1/ERG fusion proteins played similar roles. In fact, they induced oncogenic transformation of NIH3T3 cells and transcriptional repression of TGF-β receptor, whereas EWSR1/ETV1 and EWSR1/ETV4 were unable to induce oncogenic transformation in the same system. EWSR1/ETV1 and EWSR1/ETV4 in association with EWSR1/FEV play an important role in influencing the neoplasm localization. In fact, these translocations show a strong preference for extraskeletal primary sites. Their interference with important cellular pathways has also been described, for example, EWS/ETV1 is able to interact with TGF-beta pathway [29]. All three chimeric transcripts may be present in other peripheral primitive neuroectodermal tumor (pPNET) too [37, 38].

### Homeodomain transcription factors family

**Fig. 3:**
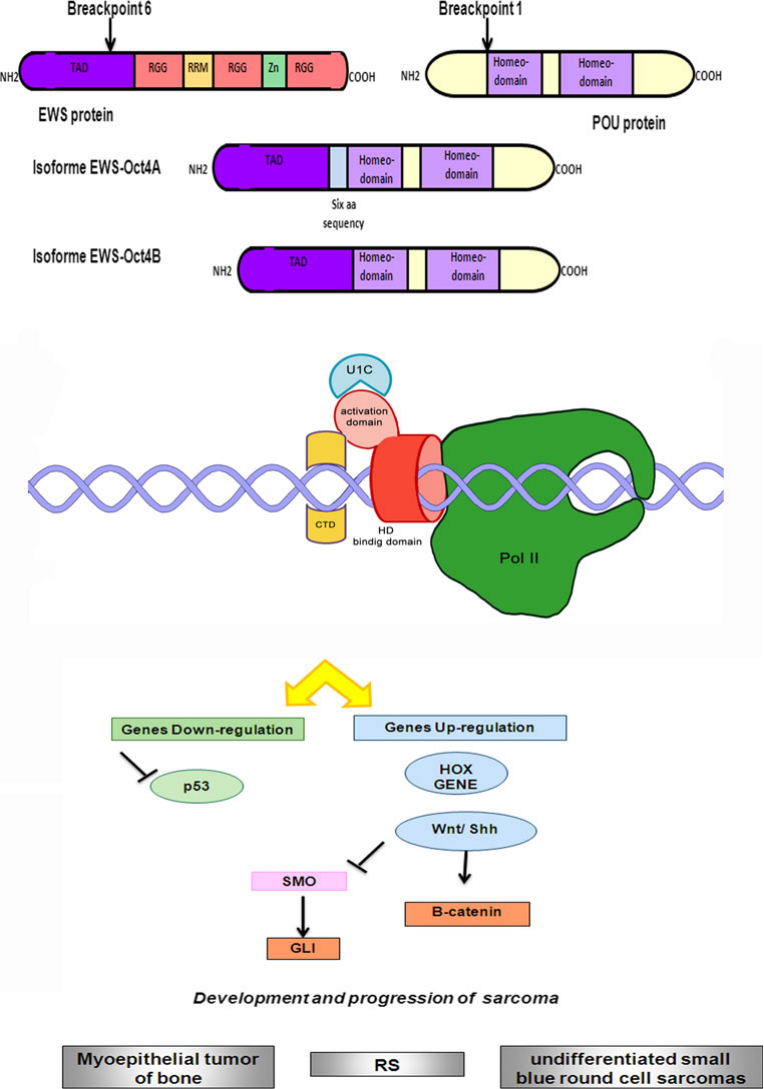
Schematic representation of EWS translocation with homeobox-genes family fusion partners: interaction with transcriptional complex on DNA and illustration of the main molecular pathways deregulated. *Gray squares* indicate the soft tissue tumors characterized by EWS/homeodomain translocations (RS: Rhabdomyosarcoma)

The homeobox gene superfamily (Fig. 3) encodes transcription factors that act as master regulators of development through their ability to activate or repress a range of downstream target genes. Numerous families exist within the homeobox gene superfamily. They are classified on the basis of the conservation of their homeodomains as well as on additional motifs that contribute to DNA binding and to the interactions with other proteins. There are many evidences, in literature, showing the strong involvement of many homeobox genes in human diseases, particularly in cancer.

#### EWSR1/POU5F1 t(6;22)(p21;q12); EWSR1/PBX1 t(1;22)(q23;q12); EWSR1/DUX4 t(4;22)(q35;q12)

POU5F1, known as OCT3/4, is a member of the POU family of transcription factors and is an important regulator of tissue-specific gene expression in lymphoid and pituitary differentiation and in early mammalian development. EWS exon 8 can translocate with POU5F1 breakpoint region, located in exon 1 [39]. EWSR1/POU5F1 translocation is described in myoepithelial tumor of bone, soft tissue, epithelium and myoepithelium [40]. Yamagunchi et al. [39] identified this type of translocation in tumor tissue derived from an undifferentiated sarcoma from the pelvic bone. Recently, using small-interfering RNA, the fundamental role of EWS-POU5F1 in tumorigenesis and tumor cell maintenance and its importance in the development and progression of sarcomas has been proved [41].

PBX1 (pre-B cell leukemia transcription factor 1) is involved in the regulation of osteogenesis and it is required for skeletal patterning and programming. A chromosomal translocation, t(1;19) involving this gene and TCF3/E2A gene is associated with pre-B cell acute lymphoblastic leukemia. EWS–PBX chimeric protein was found in myoepithelioma with two different isoforms, one in frame and one, not pathogenetically important, out of frame [42].

DUX4 (double homeobox, chromosome 4) contains two homeodomains similar in sequence to PAX3 and PAX7 homeodomains. It is involved in myogenic differentiation and cell-cycle control. EWS–DUX4 chimeric protein was found in rhabdomyosarcoma, interfering with normal muscle cell proliferation [43] and in undifferentiated small blue round cell sarcomas [44].

### Zinc finger transcription factor family

**Fig. 4:**
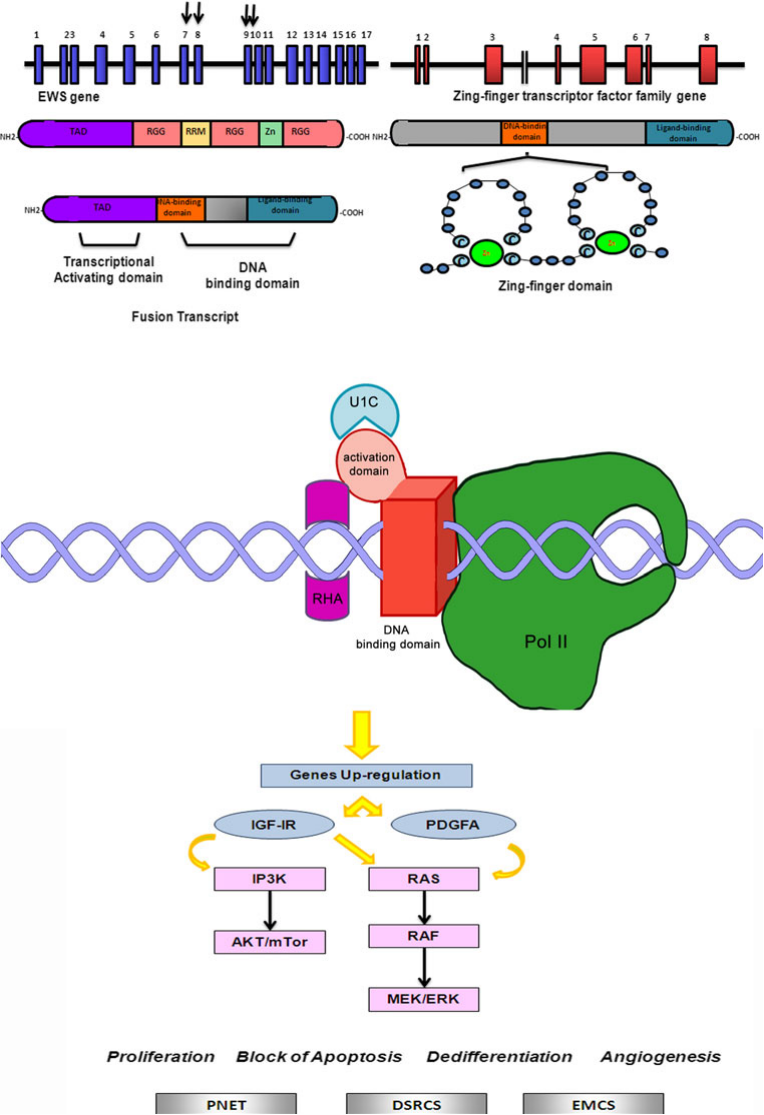
Schematic representation of EWS translocation with zinc finger-genes family fusion partners: interaction with transcriptional complex on DNA and illustration of the main molecular pathways deregulated. *Gray squares* indicate the soft tissue tumors characterized by EWS/ZN translocations (PNET: Primitive neuroectodermal tumor; DSRCS: Desmoplastic small-round-cell-sarcoma; EMCS: Extraskeletal myxoid chondrosarcomas)

Zinc finger proteins (Fig. 4) are among the most abundant proteins in eukaryotic genomes. They have a characteristic motif of 28–40 amino acids (Cys–Cys–His–His). Proteins containing zinc fingers participate in DNA recognition, RNA packaging, transcriptional activation, regulation of cell death by apoptosis, protein folding and assembly and lipid binding [45]. Some zinc finger proteins are involved in the development of human diseases, particularly in cancer [46]. EWSR1 fusion transcripts with zinc finger proteins involved always EWS exon 8 and zinc finger-genes exon 1 [47].

#### EWSR1/WT1t(11;22)(p13;q12);EWSR1/ZNF278(PATZ1)t(22;22)(q12;q12);EWSR1/ZNF384(CIZ/NMP4)t(12;22)(p13;q12); EWSR1/NR4A3 (CHN) t(9;22)(q22;q12)

*WT1* gene, deleted in individuals with Wilm’s tumor, encodes a zinc finger DNA-binding protein that acts as a transcriptional activator or repressor, mainly regulating normal formation in the genitourinary system and mesothelial tissues. The chimeric transcript EWS/WT1 was only described in desmoplastic small round cell tumor [48, 49]. EWS/WT1 is able, in this neoplasia, to induce PDGFA expression [50] and trans-activation of IGF-IR gene [51].

*Patz1* encodes a zinc finger protein responsible repression of basal transcription as well as repression of RNF4-mediated activation and transcriptional activation of c-Myc. Deregulation of Patz1 has been described in colorectal cancer [52] and in testicular tumors [53]. PATZ1 gene is located 2 Mb distal to EWS gene and is transcribed in the opposite orientation. It was described as paracentric inversion of 22q12, generating the active EWS–ZNF278 fusion gene. The chimeric transcript has been described in small round cell tumor with multidirectional differentiation rather than pPNET [47].

Transcription factor ZNF384/CIZ/NMP4 plays a role in bone metabolism and spermatogenesis. It is recurrently involved in translocations in acute lymphoblastic leukemia, where it can produce a translocation transcript with EWSR1 or with its homolog, TAF15 [54].

CHN is a member of the steroid/thyroid receptor gene superfamily, with central bipartite zinc finger DNA-binding domain. The protein is implicated in the control of cell proliferation, differentiation and apoptosis, with a prevalent expression in the central nervous system. The specific chromosomal translocation EWSR1/CHN has been observed in extrascheletric myxoid chondrosarcoma [55, 56].

### Leucine-zipper transcription factors family

**Fig. 5:**
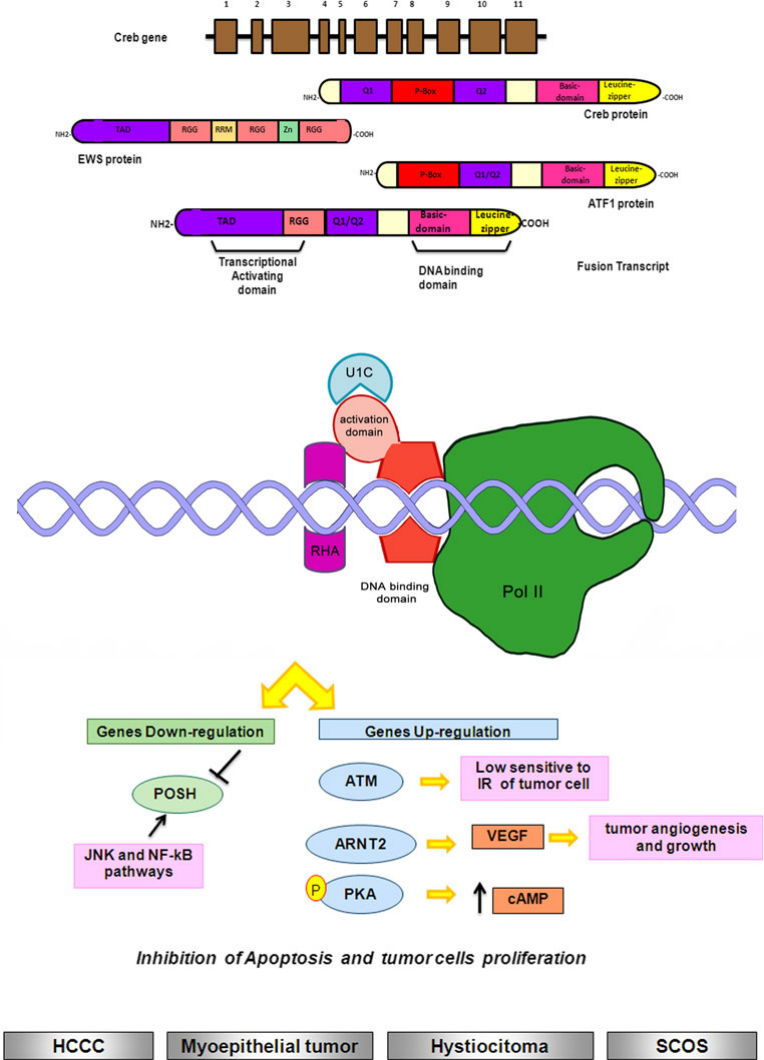
Schematic representation of EWS translocation with leucine-zipper-genes family fusion partners: interaction with transcriptional complex on DNA and illustration of the main molecular pathways deregulated. *Gray squares* indicate the soft tissue tumors characterized by EWS/leucine-zipper translocations (HCCC: Sarcomatous hepatocellular carcinoma; SCOS: Small-cell-osteo-sarcoma)

Leucine-zipper transcription factors family consists of a positively charged segment linked with a sequence of heptad repeats of leucine residues (leucine zipper) (Fig. 5). Leucine-zipper transcription factors affect several developmental processes including dendritic cell development, myeloid differentiation and brain and ocular development.

#### EWSR1/ATF1 t(12;22)(q13;q12)

ATF1 is a transcription factor (c-AMP dependent) belonging to leucine-zipper proteins. The ability of this gene to produce a fusion transcript with EWS has been demonstrated in clear-cell sarcoma, defined in the past as malignant melanoma of soft parts (MMSP) [57, 58] More recently, EWS/ATF-1 has been described in soft tissue and angiomatoid fibrous histiocytoma [59–61], and in Hyalinizing clear-cell carcinoma (HCCC), a low-grade salivary gland tumor [62]. Finally, the most recent studies have shown the presence of EWSR1–ATF1 fusion gene in a myoepithelial tumor [63] and in endobronchial pulmonary angiomatoid fibrous histiocytoma [64].

#### EWSR1/CREB1 t(2;22)(q34;q12); EWSR1/CREB3L1 t(11;22)(p11;q12)

CREB1 is a transcription factor (Fig. 5), member of the leucine-zipper family, that binds to the cAMP-responsive element. The protein is phosphorylated by several protein kinases, and induces transcription of several genes related to the cAMP pathway. Activation of CREB by phosphorylation has been implicated in the survival of mammalian cells and has been mainly involved in neuronal development. Moreover, many experimental evidences suggest that CREB1 plays a key role in mediating the malignant behavior of tumor cells. EWS is able to form a fusion transcript with CREB1 in several soft tissue tumors. It was also described as a recurrent variant fusion in clear-cell sarcomas [65, 66] and in angiomatoid fibrous histiocytoma [67]. More recently, a EWS–CREB1 translocation has been described in a case of small blue round cell tumor of the interosseous membrane [68]. Another member of cAMP response element-binding proteins is CREB3, whose activity is related to herpes simplex virus (HSV) virion protein-16 (VP16), which has been described as translocation partner of EWS in small-cell osteosarcoma. In this tumor, CREB3 is fused in frame to EWSR1 exon 11 [69].

#### EWSR1/CHOP(dITT3) t(12;22)(q13;q12)

DDIT3/CHOP is a transcription factor characterized by a carboxy-terminal region formed by a DNA-binding basic domain and a leucine-zipper dimerization domain. DDIT3 negatively regulates C/EBP heterodimers formation, preventing their binding to C/EBP sequences in the DNA. DDIT3 is strongly implicated in adipogenesis, but also in erythropoiesis and in the induction of growth arrest [70]. CHOP is mainly implicated in cytogenetically alteration that characterizes myxoid liposarcomas, in which the gene translocates with FUS gene on chromosome 16p11. However, in this tumor, CHOP could produce another fusion transcript with N-terminal part of the EWS gene [71]. Several chromosomal sites are implicated in translocation (exons 1–7 of the EWS and exons 2–4 of the CHOP gene). It was subsequently identified a novel type of EWS–CHOP fusion gene that consisted of exons 1–10 of the EWS and exons 2–4 of the CHOP gene [72].

### Other transcriptional regulators

#### EWSR1/UQCRH t(1;22)(p34;q12)

UQCRH (ubiquinol-cytochrome c reductase hinge protein) is a component of the ubiquinol-cytochrome c reductase complex associated with the mitochondrial respiratory chain, in which mediates the formation of the complex between cytochromes c and c1.

Deregulation of this gene has been described in several cancer cell lines, while a fusion transcript with EWSR1 has been detected in a panel of cancer cell lines of a small round cell sarcoma [73].

#### EWSR1/NFATC2 t(20;22)(q13;q12)

NFATC2 (nuclear factor of activated T cells, cytoplasmic, calcineurin-dependent 2) is a transcription factor implicated in control T-cell activation and function. Specifically, the transcription factor NFATc2 affects the regulation of cell differentiation and growth and plays a critical role in the development of colonic inflammation. Its deregulation was described in colon cancers [74], in pancreatic cancer [75] and in melanoma [76]. Recently, NFATc2 has been described as translocation partner of EWSR1 in Ewing sarcoma with breakpoints located in EWS exon 8 and NFATc2 exon 3 [77].

#### EWSR1/SP3 t(2;22)(q31;q12)

SP3 is a transcription factor belonging to the Sp/XKLF family able to recognize GCrich DNA motifs, found in many promoters and enhancers of housekeeping genes. Moreover, sp3 is involved in cell-cycle regulation and hormone induction. Two different breakpoints have been identified for production of EWSR1/SP3 transcripts, involving EWS exon 7 with SP3 exon 6 and EWS exon 8 with SP3 exon 6. The fusion transcripts have been detected in undifferentiated small round cell sarcomas [78].

#### EWSR1/SMARCA5 t(4;22)(q31;q12)

SMARCA5 encodes for a Helicase that possesses intrinsic ATP-dependent nucleosome-remodeling activity. The protein is mainly involved in chromatin remodeling and regulation of transcription. Its deregulation has been described in gastric cancer [79]. More recently the possibility of SMARCA5 to produce a fusion transcript in extraskeletal Ewing sarcoma/primitive neuroectodermal tumor has been described. The breakpoint involved EWS exon 7 and SMARCA5 exon 8 [80].

## Molecular techniques for EWSR1 chimeric transcripts detection

### FISH

**Fig. 6:**
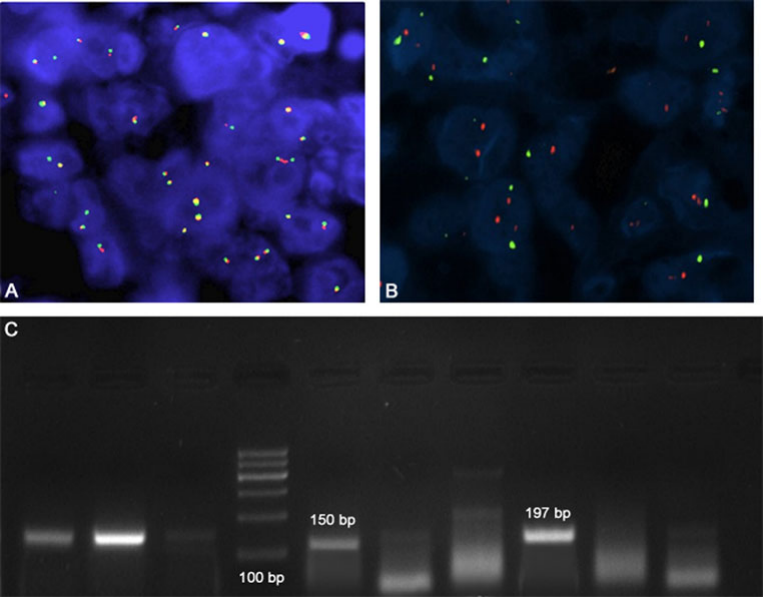
Upward, a FISH assay showing the EWS locus rearrangement with a break-apart probe: **a** absence of translocation and **b** EWS rearrangement-positive tumor cells showing one fusion, one *orange*, and one *green* signal pattern. *Arrows* shows rearrangement signals (original magnification ×60). Under, **c** a Polymerase chain reaction analysis of EWS–FLI1 translocation using EWS and FLI1 primers. Reactions were subjected to electrophoresis on a 2 % agarose gel: *Lane* 4 shows the DNA size marker (100 bp); *Lane* 1–3: Beta actin controls; *Lane* 5: Positive sample DNA (EWS/FLI1 type I-150 bp); *Lane* 8: Positive sample DNA (EWS/FLI1 type II-197 bp); *Lane* 6,7, 9 and 10: Negative samples DNA

Fluorescence in situ hybridization (FISH) is a rapid diagnostic test using molecular cytogenetic techniques. FISH technique supplements conventional cytogenetics and in some cases provides additional information, which is not detected by karyotyping. In the case of EWSR1 gene, several commercial probes has been developed by different companies (Vysis (Abbott Molecular); ARUP LABORATORIES; KREATECH DIAGNOSTIC; Cytocell Ltd.) that allow to specifically identify the breaking point in the 22q12 region of the gene (Fig. 6). However, difficulties of developing probes able to detect specific fusion partners. Since the detection of specific fusion transcripts is becoming important for prognosis and for the establishment of new therapeutic strategies, new FISH probes specific for the fusion partners of EWS have been developed. For instance, a commercial probe is able to detect the breaking of the chromosome region 11q24 related to FLI1 gene (Creative Bioarray, Abnova). However, many non-commercial probes have been developed “in-house,” by YAC and cosmid vectors, in order to detect other fusion partners of EWS, especially CREB1, FEV, WT1, ATF1. Cocktails of commercial and non-commercial probes allow to simultaneously identify translocations. Rossi et al. have developed a four-color FISH, with CREB1 proximal probe RP11-167C7 and ATF1 proximal probe RP11-189H16 labeled by nick translation with Cy5, and CREB1 distal probes G248P81788B12 and G248P89268A6 and ATF1 distal probe RP11-407N8 labeled with Cy3.

Recently, other commercial probes have been developed for detecting EWSR1/FLI1 translocation, made of a mixture of two FISH DNA probes. One of these, the Vysis LSI EWSR1 (22q12) Dual Color, Break-Apart Rearrangement Probe consists of a mixture of a ~500-kb probe labeled in SpectrumOrange, flanks the 5′ side of the EWSR1 gene and extends inward into intron 4 and a second ~1,100-kb probe labeled in SpectrumGreen, flanks the 3′ side of the EWSR1 gene (Abbott). Another FLI1/EWSR1 probe mix consists of green probes flanking the breakpoint region at the EWSR1 gene locus and red probes flanking the breakpoint region at the FLI1 locus (Cytocell Ltd.).

### Qualitative PCR

The technique more widely used for the detection of fusion transcripts is represented by RT-PCR. Many studies are reported in literature, which design specific primers for the fusion partners of EWS, able to detect all fusion transcripts. The most numerous are those associated with the detection of EWRS/FLI [23, 81–87] (Fig. 6) For ERG and ATF1, translocation partners have been also described several primers for its detection through specific RT-PCR [88–91], CREB1 [65, 66], NFTcA2 [77], WT1 [92, 93], ETV4 [94, 95] and CHOP [72, 96–98].

Finally, one of the most common problems associated with this detection technique is represented by the quality of archival biological sample, in fact, RNA fragmentation is more frequent, thus better pre-analytical procedures of sampling and storage must be developed [99]. Furthermore, the possibility of using biological material from the biobanks of fresh cryopreserved tissues has substantially resolved the problem of nucleic acids degradation [100].

### Quantitative PCR

In recent years with the advent of quantitative PCR (qPCR), many molecular diagnostic tests were oriented toward this innovative system that could provide additional quantitative information. In this technique, PCR is usually performed using primers labeled with fluorescent chromophores and a suppressor molecule. Recently, this technique has been used for the detection and quantization of the fusion transcripts, and in many cases, it is more sensitive and reliable than qualitative PCR [101, 102]. Recent reports suggest that RNA extracted from FFPE (formalin-fixed paraffin-embedded) archival materials can be successfully quantified by qRT-PCR assays, because the technique can use very small amplicons. Even in this case, the design of the probes is the main aim, because RT-PCR probes must have different characteristics and predict a particular marking which also makes them more expensive than those used for the qualitative PCR.

Different systems based on q RT-PCR have been developed for the detection of fusion transcripts in soft tumors, in particular for synovial sarcoma with Syt translocation [103–105]. A real-time RT-PCR assays specific for EWS–FLI1, EWS–ERG, EWS–ETV1, EWS–ETV4 and EWS–FEV have been recently developed [95]. However for EWSR1 translocations, only few studies have been presented in the literature.

### Western blot

There are few information about the use of western blotting for the detection of translocations in soft tissue tumors. Wang et al. [106] applied western blotting, using an antibody against the carboxyl terminal of the FLI1 protein, for the detection of the 68-kDa EWS/FLI1 fusion protein in cultured Ewing’s sarcoma cells and in four surgical biopsies of Ewing’s sarcoma. Authors stressed how this method is not dependent on the quality of mRNA in the sample and involves no risk of contamination, being a powerful complement to the reverse-transcription polymerase chain reaction (RT-PCR).

### Sequencing

Currently, sequencing represents, actually, the system for molecular investigating safer also for the detection of fusion transcripts. In fact, in most of the studies that have been carried out on the detection of EWSR1 fusion transcripts in soft tissues tumors, the sequencing of the transcript produced by PCR amplification, not only confirmed the efficiency of the investigation system used, but also the diagnosis.

## Targeting fusion transcripts

The identification of specific fusion transcripts, including several variants of spicing of EWSR1 gene could help in establishing new biotechnological systems to block their aberrant activity in cancer cells. The most modern technologies in recent years have shown the effectiveness of “silencing systems” that could be used also to establish specific target therapies for soft tissue tumors. First of all, there is an extensive literature concerning the use of siRNA in blocking fusion transcripts in several human malignancies. The use of this blocking system has also been described for numerous fusion transcripts, primarily associated with inhibition of production of protein transcripts Syt/SSX [107–110].

Even for the main fusion transcript of EWSR/FLI1, several studies have been carried out showing the effectiveness of this technique both in vitro and in vivo [111–113]. More recently, it was also described the possibility of reducing the Myc oncogene expression in Ewing sarcoma cell by siRNA targeting of EWS/FLI1 [114]. Several other systems have been developed to inhibit fusion transcript gene expression as antisense oligonucleotide nanocapsules (ODNs), whose main advantage consists in the rapid degradation, by nucleases, of ODNs. The suppression of EWS/FLI1 with nanocapsules is able to inhibit tumor growth in mice [115]. In addition to the possibility of interfering with fusion transcripts gene expression, other strategies are taking place, particularly those targeted to the construction of specific peptides against chimeric protein. It was described that EWS/FLI1 is able to bind RNA Helicase A (RHA). This link is fundamental for its oncogenic function in Ewing sarcoma. A specific small molecule, YK-4_279, can block RHA binding to EWS/FLI1, inducing apoptosis in Ewing sarcoma cells and reducing the growth of tumor cell in mice [116]. It was also described a novel peptide, defined ESAP1, able to bind EWS/FLI1 chimeric protein, with high affinity, altering cell-cycle process in Ewing sarcoma cells [117]. Finally, other alternative strategies which interfere with the activity of these transcripts are related to downstream oncogenic pathways associated with them. Regarding EWS/FLI1, it was described that this transcript can bind IGFBP3 promoter, upregulating IGF1. For this reason, therapeutic strategies targeted to inhibit IGF1 receptor pathway, as monoclonal antibody figitumumab (CP-751,871), seems to be very effective in Ewing’s sarcoma patients [118]. It was recently described that protein kinase PKC-B can be directly regulated by EWSR1/FLI1 chimeric protein. The loss of PKC-B causes apoptosis in Ewing sarcoma cells and reduces tumor growth in animal model [119]. Finally, another fusion transcript, EWSR1/CREB1, has been implicated in upregulation of MET oncogene in clear-cell sarcoma. The use of MET inhibitor ARQ197 already represents a good therapeutic strategy in clear-cell sarcoma [120].

## Conclusions

There are many different strategies to investigate EWS gene status. For diagnostic purpose, the choice of a technique relies mainly on the technological support and the “know-how” available to the pathologist. In a not too distant future, blocking strategies may be implemented at all levels, from pre-transcriptional to post-translational stages, interfering with aberrant activity of chimeric transcripts and allowing to establish new and more focused therapies for soft tissue tumors.

## References

[1] Plougastel B, Mattei MG, Thomas G, Delattre O (1994). Cloning and chromosome localization of the mouse Ews gene. Genomics.

[2] Plougastel B, Zucman J, Peter M, Thomas G, Delattre O (1993). Genomic structure of the EWS gene and its relationship to EWSR1, a site of tumor-associated chromosome translocation. Genomics.

[3] Sankar S, Lessnick SL (2011). Promiscuous partnerships in Ewing’s sarcoma. Cancer Genet.

[4] Azuma M, Embree LJ, Sabaawy H, Hickstein DD (2007). Ewing sarcoma protein EWSR1 maintains mitotic integrity and proneural cell survival in the zebrafish embryo. PLoS ONE.

[5] Rossow KL, Janknecht R (2001). The Ewing’s sarcoma gene product functions as a transcriptional activator. Cancer Res.

[6] Araya N, Hirota K, Shimamoto Y, Miyagishi M, Yoshida E, Ishida J, Kaneko S, Kaneko M, Nakajima T, Fukamizu A (2003). Cooperative interaction of EWS with CREB-binding protein selectively activates hepatocyte nuclear factor 4-mediated transcription. J Biol Chem.

[7] Gascoyne DM, Thomas GR, Latchman DS (2004). The effects of Brn-3a on neuronal differentiation and apoptosis are differentially modulated by EWS and its oncogenic derivative EWS/Fli-1. Oncogene.

[8] Lee J, Rhee BK, Bae GY, Han YM, Kim J (2005). Stimulation of Oct-4 activity by Ewing’s sarcoma protein. Stem Cells.

[9] Tannukit S, Wen X, Wang H, Paine ML (2008). TFIP11, CCNL1 and EWSR1 protein-protein interactions, and their nuclear localization. Int J Mol Sci.

[10] Alliegro MC, Alliegro MA (1996). A nuclear protein regulated during the transition from active to quiescent phenotype in cultured endothelial cells. Dev Biol.

[11] Andersson MK, Ståhlberg A, Arvidsson Y, Olofsson A, Semb H, Stenman G, Nilsson O, Aman P (2008). The multifunctional FUS, EWS and TAF15 proto-oncoproteins show cell type-specific expression patterns and involvement in cell spreading and stress response. BMC Cell Biol.

[12] Belyanskaya LL, Delattre O, Gehring H (2003). Expression and subcellular localization of Ewing sarcoma (EWS) protein is affected by the methylation process. Exp Cell Res.

[13] Leemann-Zakaryan RP, Pahlich S, Sedda MJ, Quero L, Grossenbacher D, Gehring H (2009). Dynamic subcellular localization of the Ewing sarcoma proto-oncoprotein and its association with and stabilization of microtubules. J Mol Biol.

[14] Bergeron D, Poliquin L, Kozak CA, Rassart E (1991). Identification of a common viral integration region in Cas-Br-E murine leukemia virus-induced non-T-, non-B-cell lymphomas. J Virol.

[15] Schoch C, Rieder H, Freund M, Hoelzer D, Riehm H, Fonatsch C (1995). Twenty-three cases of acute lymphoblastic leukemia with translocation t(4;11)(q21;q23): the implication of additional chromosomal aberrations. Ann Hematol.

[16] Bailly RA, Bosselut R, Zucman J, Cormier F, Delattre O, Roussel M, Thomas G, Ghysdael J (1994). DNA-binding and transcriptional activation properties of the EWS-FLI-1 fusion protein resulting from the t(11;22) translocation in Ewing sarcoma. Mol Cell Biol.

[17] Dauphinot L, De Oliveira C, Melot T, Sevenet N, Thomas V, Weissman BE, Delattre O (2001). Analysis of the expression of cell cycle regulators in Ewing cell lines: EWS-FLI-1 modulates p57KIP2 and c-Myc expression. Oncogene.

[18] Li Y, Tanaka K, Fan X, Nakatani F, Li X, Nakamura T, Takasaki M, Yamamoto S, Iwamoto Y (2010). Inhibition of the transcriptional function of p53 by EWS-Fli1 chimeric protein in Ewing family tumors. Cancer Lett.

[19] Joo J, Christensen L, Warner K, States L, Kang HG, Vo K, Lawlor ER, May WA (2009). GLI1 is a central mediator of EWS/FLI1 signaling in Ewing tumors. PLoS ONE.

[20] Li X, McGee-Lawrence ME, Decker M, Westendorf JJ (2010). The Ewing’s sarcoma fusion protein, EWS-FLI, binds Runx2 and blocks osteoblast differentiation. J Cell Biochem.

[21] Riggi N, Suvà ML, De Vito C, Provero P, Stehle JC, Baumer K, Cironi L, Janiszewska M, Petricevic T, Suvà D, Tercier S, Joseph JM, Guillou L, Stamenkovic I (2010). EWS-FLI-1 modulates miRNA145 and SOX2 expression to initiate mesenchymal stem cell reprogramming toward Ewing sarcoma cancer stem cells. Genes Dev.

[22] De Vito C, Riggi N, Suvà ML, Janiszewska M, Horlbeck J, Baumer K, Provero P, Stamenkovic I (2011). Let-7a is a direct EWS-FLI-1 target implicated in Ewing’s sarcoma development. PLoS ONE.

[23] Quezado M, Benjamin DR, Tsokos M (1997). EWS/FLI-1 fusion transcripts in three peripheral primitive neuroectodermal tumors of the kidney. Hum Pathol.

[24] Downing JR, Khandekar A, Shurtleff SA, Head DR, Parham DM, Webber BL, Pappo AS, Hulshof MG, Conn WP, Shapiro DN (1995). Multiplex RT-PCR assay for the differential diagnosis of alveolar rhabdomyosarcoma and Ewing’s sarcoma. Am J Pathol.

[25] Burchill SA, Wheeldon J, Cullinane C, Lewis IJ (1997). EWS-FLI1 fusion transcripts identified in patients with typical neuroblastoma. Eur J Cancer.

[26] Scotlandi K, Chano T, Benini S, Serra M, Manara MC, Cerisano V, Picci P, Baldini N (2000). Identification of EWS/FLI-1 transcripts in giant-cell tumor of bone. Int J Cancer.

[27] Loughran SJ, Kruse EA, Hacking DF, de Graaf CA, Hyland CD, Willson TA, Henley KJ, Ellis S, Voss AK, Metcalf D, Hilton DJ, Alexander WS, Kile BT (2008). The transcription factor Erg is essential for definitive hematopoiesis and the function of adult hematopoietic stem cells. Nat Immunol.

[28] Zoubek A, Pfleiderer C, Salzer-Kuntschik M, Amann G, Windhager R, Fink FM, Koscielniak E, Delattre O, Strehl S, Ambros PF (1994). Variability of EWS chimaeric transcripts in Ewing tumours: a comparison of clinical and molecular data. Br J Cancer.

[29] Im YH, Kim HT, Lee C, Poulin D, Welford S, Sorensen PH, Denny CT, Kim SJ (2000). EWS-FLI1, EWS-ERG, and EWS-ETV1 oncoproteins of Ewing tumor family all suppress transcription of transforming growth factor beta type II receptor gene. Cancer Res.

[30] Matsui Y, Chansky HA, Barahmand-Pour F, Zielinska-Kwiatkowska A, Tsumaki N, Myoui A, Yoshikawa H, Yang L, Eyre DR (2003). COL11A2 collagen gene transcription is differentially regulated by EWS/ERG sarcoma fusion protein and wild-type ERG. J Biol Chem.

[31] Irifune H, Nishimori H, Watanabe G, Yoshida K, Ikeda T, Matsui C, Morohashi M, Kawaguchi S, Nagoya S, Wada T, Yamashita T, Nakamura Y, Tokino T (2005). Aberrant laminin beta3 isoforms downstream of EWS-ETS fusion genes in Ewing family tumors. Cancer Biol Ther.

[32] Minoletti F, Sozzi G, Tornielli S, Pilotti S, Azzarelli A, Pierotti MA, Radice P (1998). A novel EWS-ERG rearrangement generating two hybrid mRNAs in a peripheral primitive neuroectodermal tumour (pPNET) with a t(15;22) translocation. J Pathol.

[33] Ordi J, de Alava E, Torné A, Mellado B, Pardo-Mindan J, Iglesias X, Cardesa A (1998). Intraabdominal desmoplastic small round cell tumor with EWS/ERG fusion transcript. Am J Surg Pathol.

[34] Peter M, Couturier J, Pacquement H, Michon J, Thomas G, Magdelenat H, Delattre O (1997). A new member of the ETS family fused to EWS in Ewing tumors. Oncogene.

[35] Jeon IS, Davis JN, Braun BS, Sublett JE, Roussel MF, Denny CT, Shapiro DN (1995). A variant Ewing’s sarcoma translocation (7;22) fuses the EWS gene to the ETS gene ETV1. Oncogene.

[36] Ishida S, Yoshida K, Kaneko Y, Tanaka Y, Sasaki Y, Urano F, Umezawa A, Hata J, Fujinaga K (1998). The genomic breakpoint and chimeric transcripts in the EWSR1-ETV4/E1AF gene fusion in Ewing sarcoma. Cytogenet Cell Genet.

[37] Teitell MA, Thompson AD, Sorensen PH, Shimada H, Triche TJ, Denny CT (1999). EWS/ETS fusion genes induce epithelial and neuroectodermal differentiation in NIH3T3 fibroblasts. Lab Invest.

[38] Llombart-Bosch A, Pellín A, Carda C, Noguera R, Navarro S, Peydró-Olaya A (2000). Soft tissue Ewing sarcoma–peripheral primitive neuroectodermal tumor with atypical clear cell pattern shows a new type of EWS-FEV fusion transcript. Diagn Mol Pathol.

[39] Yamaguchi S, Yamazaki Y, Ishikawa Y, Kawaguchi N, Mukai H, Nakamura T (2005). EWSR1 is fused to POU5F1 in a bone tumor with translocation t(6;22)(p21;q12). Genes Chromosomes Cancer.

[40] Antonescu CR, Zhang L, Chang NE, Pawel BR, Travis W, Katabi N, Edelman M, Rosenberg AE, Nielsen GP (2010). Dal Cin P, Fletcher CD. EWSR1-POU5F1 fusion in soft tissue myoepithelial tumors. A molecular analysis of sixty-six cases, including soft tissue, bone, and visceral lesions, showing common involvement of the EWSR1 gene. Genes Chromosomes Cancer.

[41] Fujino T, Nomura K, Ishikawa Y, Makino H, Umezawa A, Aburatani H, Nagasaki K, Nakamura T (2010). Function of EWS-POU5F1 in sarcomagenesis and tumor cell maintenance. Am J Pathol.

[42] Brandal P, Panagopoulos I, Bjerkehagen B, Gorunova L, Skjeldal S, Micci F, Heim S (2008). Detection of a t(1;22)(q23;q12) translocation leading to an EWSR1-PBX1 fusion gene in a myoepithelioma. Genes Chromosomes Cancer.

[43] Sirvent N, Trassard M, Ebran N, Attias R, Pedeutour F (2009). Fusion of EWSR1 with the DUX4 facioscapulohumeral muscular dystrophy region resulting from t(4;22)(q35;q12) in a case of embryonal rhabdomyosarcoma. Cancer Genet Cytogenet.

[44] Italiano A, Sung YS, Zhang L, Singer S, Maki RG, Coindre JM, Antonescu CR (2012). High prevalence of CIC fusion with double-homeobox (DUX4) transcription factors in EWSR1-negative undifferentiated small blue round cell sarcomas. Genes Chromosomes Cancer.

[45] Laity JH, Lee BM, Wright PE (2001). Zinc finger proteins: new insights into structural and functional diversity. Curr Opin Struct Biol.

[46] Ladomery M, Dellaire G (2002). Multifunctional zinc finger proteins in development and disease. Ann Hum Genet.

[47] Mastrangelo T, Modena P, Tornielli S, Bullrich F, Testi MA, Mezzelani A, Radice P, Azzarelli A, Pilotti S, Croce CM, Pierotti MA, Sozzi G (2000). A novel zinc finger gene is fused to EWS in small round cell tumor. Oncogene.

[48] Gerald WL, Rosai J, Ladanyi M (1995). Characterization of the genomic breakpoint and chimeric transcripts in the EWS-WT1 gene fusion of desmoplastic small round cell tumor. Proc Natl Acad Sci USA.

[49] Shimizu Y, Mitsui T, Kawakami T, Ikegami T, Kanazawa C, Katsuura M, Obata K, Yamagiwa I, Hayasaka K (1998). Novel breakpoints of the EWS gene and the WT1 gene in a desmoplastic small round cell tumor. Cancer Genet Cytogenet.

[50] Lee SB, Kolquist KA, Nichols K, Englert C, Maheswaran S, Ladanyi M, Gerald WL, Haber DA (1997). The EWS-WT1 translocation product induces PDGFA in desmoplastic small round-cell tumour. Nat Genet.

[51] Werner H, Idelman G, Rubinstein M, Pattee P, Nagalla SR, Roberts CT (2007). A novel EWS-WT1 gene fusion product in desmoplastic small round cell tumor is a potent transactivator of the insulin-like growth factor-I receptor (IGF-IR) gene. Cancer Lett.

[52] Tian X, Sun D, Zhang Y, Zhao S, Xiong H, Fang J (2008). Zinc finger protein 278, a potential oncogene in human colorectal cancer. Acta Biochim Biophys Sin (Shanghai).

[53] Fedele M, Franco R, Salvatore G, Paronetto MP, Barbagallo F, Pero R, Chiariotti L, Sette C, Tramontano D, Chieffi G, Fusco A, Chieffi P (2008). PATZ1 gene has a critical role in the spermatogenesis and testicular tumours. J Pathol.

[54] Martini A, La Starza R, Janssen H, Bilhou-Nabera C, Corveleyn A, Somers R, Aventin A, Foà R, Hagemeijer A, Mecucci C, Marynen P (2002). Recurrent rearrangement of the Ewing’s sarcoma gene, EWSR1, or its homologue, TAF15, with the transcription factor CIZ/NMP4 in acute leukemia. Cancer Res.

[55] Clark J, Benjamin H, Gill S, Sidhar S, Goodwin G, Crew J, Gusterson BA, Shipley J, Cooper CS (1996). Fusion of the EWS gene to CHN, a member of the steroid/thyroid receptor gene superfamily, in extrascheletric myxoid chondrosarcoma. Oncogene.

[56] Brody RI, Ueda T, Hamelin A, Jhanwar SC, Bridge JA, Healey JH, Huvos AG, Gerald WL, Ladanyi M (1997). Molecular analysis of the fusion of EWS to an orphan nuclear receptor gene in extraskeletal myxoid chondrosarcoma. Am J Pathol.

[57] Zucman J, Delattre O, Desmaze C, Epstein AL, Stenman G, Speleman F, Fletchers CDM, Aurias A, Thomas G (1993). EWS and ATF-1 gene fusion induced by t(12;22) translocation in malignant melanoma of soft parts. Nat Genet.

[58] Hiraga H, Nojima T, Abe S, Yamashiro K, Yamawaki S, Kaneda K, Nagashima K (1997). Establishment of a new continuous clear cell sarcoma cell line. Morphological and cytogenetic characterization and detection of chimaeric EWS/ATF-1 transcripts. Virchows Arch.

[59] Hallor KH, Mertens F, Jin Y, Meis-Kindblom JM, Kindblom LG, Behrendtz M, Kalén A, Mandahl N, Panagopoulos I (2005). Fusion of the EWSR1 and ATF1 genes without expression of the MITF-M transcript in angiomatoid fibrous histiocytoma. Genes Chromosomes Cancer.

[60] Hallor KH, Micci F, Meis-Kindblom JM, Kindblom LG, Bacchini P, Mandahl N, Mertens F, Panagopoulos I (2007). Fusion genes in angiomatoid fibrous histiocytoma. Cancer Lett.

[61] Rossi S, Szuhai K, Ijszenga M, Tanke HJ, Zanatta L, Sciot R, Fletcher CD (2007). Dei Tos AP, Hogendoorn PC. EWSR1-CREB1 and EWSR1-ATF1 fusion genes in angiomatoid fibrous histiocytoma. Clin Cancer Res.

[62] Antonescu CR, Katabi N, Zhang L, Sung YS, Seethala RR, Jordan RC, Perez-Ordoñez B, Have C, Asa SL, Leong IT, Bradley G, Klieb H (2011). Weinreb I EWSR1-ATF1 fusion is a novel and consistent finding in Hyalinizing clear-cell carcinoma of salivary gland. Genes Chromosomes Cancer.

[63] Flucke U, Mentzel T, Verdijk MA, Slootweg PJ, Creytens DH, Suurmeijer AJ, Tops BB (2012). EWSR1-ATF1 chimeric transcript in a myoepithelial tumor of soft tissue: a case report. Hum Pathol.

[64] Thway K, Nicholson AG, Wallace WA, Al-Nafussi A, Pilling J, Fisher C (2012). Endobronchial pulmonary angiomatoid fibrous histiocytoma: two cases with EWSR1-CREB1 and EWSR1-ATF1 fusions. Am J Surg Pathol.

[65] Antonescu CR, Nafa K, Segal NH (2006). Dal Cin P, Ladanyi M. EWS-CREB1: a recurrent variant fusion in clear cell sarcoma–association with gastrointestinal location and absence of melanocytic differentiation. Clin Cancer Res.

[66] Wang WL, Mayordomo E, Zhang W, Hernandez VS, Tuvin D, Garcia L, Lev DC, Lazar AJ, López-Terrada D (2009). Detection and characterization of EWSR1/ATF1 and EWSR1/CREB1 chimeric transcripts in clear cell sarcoma (melanoma of soft parts). Mod Pathol.

[67] Antonescu CR (2007). Dal Cin P, Nafa K, Teot LA, Surti U, Fletcher CD, Ladanyi M. EWSR1-CREB1 is the predominant gene fusion in angiomatoid fibrous histiocytoma. Genes Chromosomes Cancer.

[68] Pacheco M, Horsman DE, Hayes MM, Clarkson PW, Huwait H, Nielsen TO (2010). Small blue round cell tumor of the interosseous membrane bearing a t(2;22)(q34;q12)/EWS-CREB1 translocation: a case report. Mol Cytogenet.

[69] Debelenko LV, McGregor LM, Shivakumar BR, Dorfman HD, Raimondi SC (2011). A novel EWSR1-CREB3L1 fusion transcript in a case of small cell osteosarcoma. Genes Chromosomes Cancer.

[70] Batchvarova N, Wang XZ, Ron D (1995). Inhibition of adipogenesis by the stress-induced protein CHOP (Gadd153). EMBO J.

[71] Panagopoulos I, Höglund M, Mertens F, Mandahl N, Mitelman F, Aman P (1996). Fusion of the EWS and CHOP genes in myxoid liposarcoma. Oncogene.

[72] Hosaka T, Nakashima Y, Kusuzaki K, Murata H, Nakayama T, Nakamata T, Aoyama T, Okamoto T, Nishijo K, Araki N, Tsuboyama T, Nakamura T, Toguchida J (2002). A novel type of EWS-CHOP fusion gene in two cases of myxoid liposarcoma. J Mol Diagn.

[73] Modena P, Testi MA, Facchinetti F, Mezzanzanica D, Radice MT, Pilotti S, Sozzi G (2003). UQCRH gene encoding mitochondrial hinge protein is interrupted by a translocation in a soft-tissue sarcoma and epigenetically inactivated in some cancer cell lines. Oncogene.

[74] Gerlach K, Daniel C, Lehr HA, Nikolaev A, Gerlach T, Atreya R, Rose-John S, Neurath MF, Weigmann B (2012). Transcription factor NFATc2 controls the emergence of colon cancer associated with IL-6-dependent colitis. Cancer Res.

[75] Baumgart S, Glesel E, Singh G, Chen NM, Reutlinger K, Zhang J, Billadeau DD, Fernandez-Zapico ME, Gress TM, Singh SK, Ellenrieder V (2012). Restricted heterochromatin formation links NFATc2 repressor activity with growth promotion in pancreatic cancer. Gastroenterology.

[76] Perotti V, Baldassari P, Bersani I, Molla A, Vegetti C, Tassi E, Col JD, Dolcetti R, Anichini A, Mortarini R (2012). NFATc2 is a potential therapeutic target in human melanoma. J Invest Dermatol.

[77] Szuhai K, Ijszenga M, de Jong D, Karseladze A, Tanke HJ, Hogendoorn PC (2009). The NFATc2 gene is involved in a novel cloned translocation in a Ewing sarcoma variant that couples its function in immunology to oncology. Clin Cancer Res.

[78] Wang L, Bhargava R, Zheng T, Wexler L, Collins MH, Roulston D, Ladanyi M (2007). Undifferentiated small round cell sarcomas with rare EWS gene fusions: identification of a novel EWS-SP3 fusion and of additional cases with the EWS-ETV1 and EWS-FEV fusions. J Mol Diagn.

[79] Gigek CO, Lisboa LC, Leal MF, Silva PN, Lima EM, Khayat AS, Assumpção PP, Burbano RR, Smith Mde A (2011). SMARCA5 methylation and expression in gastric cancer. Cancer Invest.

[80] Sumegi J, Nishio J, Nelson M, Frayer RW, Perry D, Bridge JA (2011). A novel t(4;22)(q31;q12) produces an EWSR1-SMARCA5 fusion in extraskeletal Ewing sarcoma/primitive neuroectodermal tumor. Mod Pathol.

[81] Meier VS, Kühne T, Jundt G, Gudat F (1998). Molecular diagnosis of Ewing tumors: improved detection of EWS-FLI-1 and EWS-ERG chimeric transcripts and rapid determination of exon combinations. Diagn Mol Pathol.

[82] Kawauchi S, Fukuda T, Miyamoto S, Yoshioka J, Shirahama S, Saito T, Tsukamoto N (1998). Peripheral primitive neuroectodermal tumor of the ovary confirmed by CD99 immunostaining, karyotypic analysis, and RT-PCR for EWS/FLI-1 chimeric mRNA. Am J Surg Pathol.

[83] Hisaoka M, Tsuji S, Morimitsu Y, Hashimoto H, Shimajiri S, Komiya S, Ushijima M (1999). Molecular detection of EWS-FLI1 chimeric transcripts in Ewing family tumors by nested reverse transcription-polymerase chain reaction: application to archival paraffin-embedded tumor tissues. APMIS.

[84] Naito N, Kawai A, Ouchida M, Dan’ura T, Morimoto Y, Ozaki T, Shimizu K, Inoue H (2000). A reverse transcriptase-polymerase chain reaction assay in the diagnosis of soft tissue sarcomas. Cancer.

[85] Tokudome N, Tanaka K, Kai MH, Sueyoshi K, Matsukita S, Setoguchi T (2002). Primitive neuroectodermal tumor of the transverse colonic mesentery defined by the presence of EWS-FLI1 chimeric mRNA in a Japanese woman. J Gastroenterol.

[86] Stegmaier S, Leuschner I, Aakcha-Rudel E, Münch P, Kazanowska B, Bekassy A, Treuner J, Koscielniak E (2004). Identification of various exon combinations of the ews/fli1 translocation: an optimized RT-PCR method for paraffin embedded tissue a report by the CWS-study group. Klin Padiatr.

[87] Qian X, Jin L, Shearer BM, Ketterling RP, Jalal SM, Lloyd RV (2005). Molecular diagnosis of Ewing’s sarcoma/primitive neuroectodermal tumor in formalin-fixed paraffin-embedded tissues by RT-PCR and fluorescence in situ hybridization. Diagn Mol Pathol.

[88] Pellín A, Monteagudo C, López-Ginés C, Carda C, Boix J, Llombart-Bosch A (1998). New type of chimeric fusion product between the EWS and ATFI genes in clear cell sarcoma (malignant melanoma of soft parts). Genes Chromosomes Cancer.

[89] Antonescu CR, Tschernyavsky SJ, Woodruff JM, Jungbluth AA, Brennan MF, Ladanyi M (2002). Molecular diagnosis of clear cell sarcoma: detection of EWS-ATF1 and MITF-M transcripts and histopathological and ultrastructural analysis of 12 cases. J Mol Diagn.

[90] Covinsky M, Gong S, Rajaram V, Perry A, Pfeifer J (2005). EWS-ATF1 fusion transcripts in gastrointestinal tumors previously diagnosed as malignant melanoma. Hum Pathol.

[91] Curry CV, Dishop MK, Hicks MJ, Naeem R, Reed JA, López-Terrada DH (2008). Clear cell sarcoma of soft tissue: diagnostic utility of fluorescence in situ hybridization and reverse transcriptase polymerase chain reaction. J Cutan Pathol.

[92] Hamazaki M, Okita H, Hata J, Shimizu S, Kobayashi H, Aoki K, Nara T (2006). Desmoplastic small cell tumor of soft tissue: molecular variant of EWS-WT1 chimeric fusion. Pathol Int.

[93] Gautam U, Srinivasan R, Rajwanshi A, Bansal D, Marwaha RK, Vasishtha RK (2010). Reverse transcriptase-polymerase chain reaction as an ancillary molecular technique in the diagnosis of small blue round cell tumors by fine-needle aspiration cytology. Am J Clin Pathol.

[94] Rougemont AL, Bouron-Dal Soglio D, Patey-Mariaud de Serre N, Fetni R, Fan L, Barrette S, Fournet JC (2012). A t(17;22)(q21;q12) with partial ETV4 deletion in a soft tissue Ewing sarcoma. Cancer Genet.

[95] Lewis TB, Coffin CM, Bernard PS (2007). Differentiating Ewing’s sarcoma from other round blue cell tumors using a RT-PCR translocation panel on formalin-fixed paraffin-embedded tissues. Mod Pathol.

[96] Dal Cin P, Sciot R, Panagopoulos I, Aman P, Samson I, Mandahl N (1997). Additional evidence of a variant translocation t(12;22) with EWS/CHOP fusion in myxoid liposarcoma: clinicopathological features. J Pathol.

[97] Matsui Y, Ueda T, Kubo T, Hasegawa T, Tomita Y, Okamoto M (2006). A novel type of EWS-CHOP fusion gene in myxoid liposarcoma. Biochem Biophys Res Commun.

[98] Suzuki K, Matsui Y, Endo K, Kubo T, Hasegawa T, Kimura T (2010). Myxoid liposarcoma with EWS-CHOP type 1 fusion gene. Anticancer Res.

[99] Bohmann K, Hennig G, Rogel U, Poremba C, Mueller BM, Fritz P (2009). RNA extraction from archival formalin-fixed paraffin-embedded tissue: a comparison of manual, semiautomated, and fully automated purification methods. Clin Chem.

[100] Botti G, Franco R, Cantile M, Ciliberto G, Ascierto PA (2012). Tumor biobanks in translational medicine. J Transl Med.

[101] Guo JQ, Lin H, Kantarjian H, Talpaz M, Champlin R, Andreeff M (2002). Comparison of competitive-nested PCR and real-time PCR in detecting BCR-ABL fusion transcripts in chronic myeloid leukemia patients. Leukemia.

[102] Wattjes MP, Krauter J, Nagel S, Heidenreich O, Ganser A, Heil G (2000). Comparison of nested competitive RT-PCR and real-time RT-PCR for the detection and quantification of AML1/MTG8 fusion transcripts in t(8;21) positive acute myelogenous leukemia. Leukemia.

[103] Bijwaard KE, Fetsch JF, Przygodzki R, Taubenberger JK, Lichy JH (2002). Detection of SYT-SSX fusion transcripts in archival synovial sarcomas by real-time reverse transcriptase-polymerase chain reaction. J Mol Diagn.

[104] Hostein I, Menard A, Bui BN, Lussan C, Wafflart J, Delattre O (2002). Molecular detection of the synovial sarcoma translocation t(X;18) by real-time polymerase chain reaction in paraffin-embedded material. Diagn Mol Pathol.

[105] Nikiforova MN, Groen P, Mutema G, Nikiforov YE, Witte D (2005). Detection of SYT-SSX rearrangements in synovial sarcomas by real-time one-step RT-PCR. Pediatr Dev Pathol.

[106] Wang M, Nilsson G, Carlberg M, Dricu A, Wejde J, Kreicbergs A (1998). Specific and sensitive detection of the EWS/FLI1 fusion protein in Ewing’s sarcoma by western blotting. Virchows Arch.

[107] Peng C, Guo W, Yang Y, Zhao H (2008). Downregulation of SS18-SSX1 expression by small interfering RNA inhibits growth and induces apoptosis in human synovial sarcoma cell line HS-SY-II in vitro. Eur J Cancer Prev.

[108] Takenaka S, Naka N, Araki N, Hashimoto N, Ueda T, Yoshioka K (2010). Downregulation of SS18-SSX1 expression in synovial sarcoma by small interfering RNA enhances the focal adhesion pathway and inhibits anchorage-independent growth in vitro and tumor growth in vivo. Int J Oncol.

[109] Oikawa K, Tanaka M, Itoh S, Takanashi M, Ozaki T, Muragaki Y (2012). A novel oncogenic pathway by TLS-CHOP involving repression of MDA-7/IL-24 expression. Br J Cancer.

[110] Göransson M, Elias E, Ståhlberg A, Olofsson A, Andersson C, Aman P (2005). Myxoid liposarcoma FUS-DDIT3 fusion oncogene induces C/EBP beta-mediated interleukin 6 expression. Int J Cancer.

[111] Chansky HA, Barahmand-Pour F, Mei Q, Kahn-Farooqi W, Zielinska-Kwiatkowska A, Blackburn M, Chansky K, Conrad EU, Bruckner JD, Greenlee TK, Yang L (2004). Targeting of EWS/FLI-1 by RNA interference attenuates the tumor phenotype of Ewing’s sarcoma cells in vitro. J Orthop Res.

[112] Prieur A, Tirode F, Cohen P, Delattre O (2004). EWS/FLI-1 silencing and gene profiling of Ewing cells reveal downstream oncogenic pathways and a crucial role for repression of insulin-like growth factor binding protein 3. Mol Cell Biol.

[113] Hu-Lieskovan S, Heidel JD, Bartlett DW, Davis ME, Triche TJ (2005). Sequence-specific knockdown of EWS-FLI1 by targeted, nonviral delivery of small interfering RNA inhibits tumor growth in a murine model of metastatic Ewing’s sarcoma. Cancer Res.

[114] Takigami I, Ohno T, Kitade Y, Hara A, Nagano A, Kawai G, Saitou M, Matsuhashi A, Yamada K, Shimizu K (2011). Synthetic siRNA targeting the breakpoint of EWS/Fli-1 inhibits growth of Ewing sarcoma xenografts in a mouse model. Int J Cancer.

[115] Toub N, Bertrand JR, Malvy C, Fattal E, Couvreur P (2006). Antisense oligonucleotide nanocapsules efficiently inhibit EWS-Fli1 expression in a Ewing’s sarcoma model. Oligonucleotides.

[116] Erkizan HV, Kong Y, Merchant M, Schlottmann S, Barber-Rotenberg JS, Yuan L, Abaan OD, Chou TH, Dakshanamurthy S, Brown ML, Uren A, Toretsky JA (2009). A small molecule blocking oncogenic protein EWS-FLI1 interaction with RNA helicase a inhibits growth of Ewing’s sarcoma. Nat Med.

[117] Erkizan HV, Scher LJ, Gamble SE, Barber-Rotenberg JS, Sajwan KP, Üren A, Toretsky JA (2011). Novel peptide binds EWS-FLI1 and reduces the oncogenic potential in Ewing tumors. Cell Cycle.

[118] Olmos D, Postel-Vinay S, Molife LR, Okuno SH, Schuetze SM, Paccagnella ML, Batzel GN, Yin D, Pritchard-Jones K, Judson I, Worden FP, Gualberto A, Scurr M, de Bono JS, Haluska P (2010). Safety, pharmacokinetics, and preliminary activity of the anti-IGF-1R antibody figitumumab (CP-751,871) in patients with sarcoma and Ewing’s sarcoma: a phase 1 expansion cohort study. Lancet Oncol.

[119] Surdez D, Benetkiewicz M, Perrin V, Han ZY, Pierron G, Ballet S, Lamoureux F, Rédini F, Decouvelaere AV, Daudigeos-Dubus E, Geoerger B, de Pinieux G, Delattre O, Tirode F (2012). Targeting the EWSR1-FLI1 oncogene-induced protein kinase PKC-β abolishes Ewing sarcoma growth. Cancer Res.

[120] Davis IJ, McFadden AW, Zhang Y, Coxon A, Burgess TL, Wagner AJ, Fisher DE (2010). Identification of the receptor tyrosine kinase c-Met and its ligand, hepatocyte growth factor, as therapeutic targets in clear cell sarcoma. Cancer Res.

